# ASSOCIATION OF BEHAVIORAL AND PSYCHOLOGICAL SYMPTOMS OF DEMENTIA WITH ADVERSE OUTCOMES POSTHOSPITALIZATION

**DOI:** 10.1093/geroni/igad104.0305

**Published:** 2023-12-21

**Authors:** Diane Berish

**Affiliations:** The Pennsylvania State University, University Park, Pennsylvania, United States

## Abstract

In hospitalized persons with dementia, behavioral and psychological symptoms of dementia (BPSD), also known as behavioral symptoms of distress, are associated with increased morbidity, long-term care admissions, and reduced care partner well-being. The Fam-FFC study provided the opportunity to examine the association between BPSD severity and adverse, post-acute outcomes (falls, emergency department transfers, hospital admissions). Logistic regression analysis examined the association of behavioral symptoms (Neuropsychiatric Inventory Questionnaire, NPI) and odds of having a fall, emergency department transfer, and hospital admission, controlling for the intervention, and caregiver and patient characteristics. NPI scores decreased from admission (M = 7.81, SD = 6.12), to discharge (M = 6.4, SD = 5.93), to two months (M = 5.38, SD = 5.91), to six-month follow-up (M = 5.08, SD = 5.94). Higher admission NPI scores were associated with greater odds of experiencing at least one fall (OR = 1.067, p = 0.042) by discharge. Higher discharge NPI scores were associated with higher odds of ED transfer (OR = 1.062, p = 0.006) and at least one hospitalization (OR = 1.06, p = 0.008) after two months. Higher NPI scores at 2-month follow-up were associated with increased odds of ED transfer (OR = 1.062, p = 0.004), experiencing at least one injury (OR = 1.055, p = 0.031), falls (OR = 1.056, p = 0.012), and hospitalizations (OR = 1.065, p = 0.004) at six months. Results underscore the importance of providing interventions to reduce BPSD as a potentially modifiable risk factor to reduce adverse outcomes.

